# Development of a customizable mouse backbone spectral flow cytometry panel to delineate immune cell populations in normal and tumor tissues

**DOI:** 10.3389/fimmu.2024.1374943

**Published:** 2024-03-27

**Authors:** Ana Leda F. Longhini, Inés Fernández-Maestre, Margaret C. Kennedy, Matthew G. Wereski, Shoron Mowla, Wenbin Xiao, Scott W. Lowe, Ross L. Levine, Rui Gardner

**Affiliations:** ^1^ Flow Cytometry Core Facility, Memorial Sloan Kettering Cancer Center (MSKCC), New York, NY, United States; ^2^ Memorial Sloan Kettering Cancer Center, New York, NY, United States; ^3^ Louis V. Gerstner Jr Graduate School of Biomedical Sciences, Memorial Sloan Kettering Cancer Center, New York, NY, United States; ^4^ Department of Cancer Biology and Genetics, Memorial Sloan Kettering Cancer Center, New York, NY, United States; ^5^ Center for Hematologic Malignancies, Memorial Sloan Kettering Cancer Center, New York, NY, United States; ^6^ Department of Pathology and Laboratory Medicine, Hematopathology Service, Memorial Sloan Kettering Cancer Center, New York, NY, United States; ^7^ Howard Hughes Medical Institute, Memorial Sloan Kettering Cancer Center, New York, NY, United States; ^8^ Department of Medicine, Leukemia Service, Memorial Sloan Kettering Cancer Center, New York, NY, United States

**Keywords:** tumor microenvironment (TME), backbone panel, immune cells, spectral flow cytometry, mouse, immunophenotyping

## Abstract

**Introduction:**

*In vivo* studies of cancer biology and assessment of therapeutic efficacy are critical to advancing cancer research and ultimately improving patient outcomes. Murine cancer models have proven to be an invaluable tool in pre-clinical studies. In this context, multi-parameter flow cytometry is a powerful method for elucidating the profile of immune cells within the tumor microenvironment and/or play a role in hematological diseases. However, designing an appropriate multi-parameter panel to comprehensively profile the increasing diversity of immune cells across different murine tissues can be extremely challenging.

**Methods:**

To address this issue, we designed a panel with 13 fixed markers that define the major immune populations –referred to as the backbone panel– that can be profiled in different tissues but with the option to incorporate up to seven additional fluorochromes, including any marker specific to the study in question.

**Results:**

This backbone panel maintains its resolution across different spectral flow cytometers and organs, both hematopoietic and non-hematopoietic, as well as tumors with complex immune microenvironments.

**Discussion:**

Having a robust backbone that can be easily customized with pre-validated drop-in fluorochromes saves time and resources and brings consistency and standardization, making it a versatile solution for immuno-oncology researchers. In addition, the approach presented here can serve as a guide to develop similar types of customizable backbone panels for different research questions requiring high-parameter flow cytometry panels.

## 1 Introduction

Studying murine cancer models is critical for comprehending the biological mechanisms of cancer development and the effectiveness of potential therapies *in vivo* (1). Despite advances in *ex vivo* organoid culture research, murine models still provide a more accurate depiction of the natural tumor microenvironment (TME) and aid in untangling the complexities of cancer pathogenesis. Unlike 2-D *in vitro* cultures, which are too simplistic to mimic the tumor-specific architecture, and 3-D models, which show high variability and lack of a native microenvironment and recruitment of immune cells, murine models offer a natural TME representation (2–7). Particularly, syngeneic models preserve the tumor architecture and the relative proportion of cancer and stromal cells, including in the context of orthotopic transplantation, which leads to a microenvironment more similar to human cancer (1, 8, 9).

The TME is a highly complex and dynamic ecosystem known to regulate tumorigenesis, cancer progression, and drug resistance; and its composition differs depending on tumor type and location. Immune cells are a significant component of the microenvironment in both solid tumors and hematological malignancies and play a fundamental role in determining cancer cell fate, metastatic capacity, and disease progression. Cancer cells can recruit and interact with various immune cells, including macrophages, polymorphonuclear cells, mast cells, natural killer cells, dendritic cells, and T and B lymphocytes (10–13). Therefore, myeloid and lymphoid cells can have both protumor and antitumor effects, making it essential to understand the relative contribution of each immune cell subset to the TME and the infiltrated organs, or the observed phenotype and/or response to immunotherapy (14–18). Yet, studying immune cell populations in the TME can pose challenges due to its dynamic nature, the unique characteristics of the affected organ or tissue, and the tumor-specific recruitment of myeloid and lymphoid populations (11, 19, 20).

At the single-cell level, flow cytometry is an effective tool for characterizing immune cell phenotypes in a variety of situations, including solid tumors, hematological malignancies, minimal residual disease, and metastatic progression (21–25). While polychromatic –also referred to as conventional– flow cytometry is commonly used to assess hematological disease in mice (26–29), single cell sequencing has emerged as an alternative approach for immune cell profiling of solid tumors in the recent years (30, 31). This is due to the challenges of polychromatic flow cytometry experiments, where the number of parameters is limited by the number of detectors in the cytometer. This constraint can also restrict the study of marker co-expression in different cell populations, often requiring different tubes for multiple panels, which may not always be feasible due to sample scarcity (32–35). Spectral flow cytometry addresses these issues by allowing greater flexibility in panel design and facilitating the acquisition of higher dimensional data (32). However, current multispectral flow cytometry techniques still face challenges in analyzing diverse tissue types, even when comparing tumor tissue to its non-tumor counterpart, which may require the assignment of their autofluorescence as a separate fluorochrome and additional spectral unmixing (36, 37).

Since 2022, a few multiparameter panels for spectral cytometers have been introduced to study subtypes of immune cells on murine samples (38–43). Although some of the panels include markers for both myeloid and lymphoid populations, they were optimized for a specific type of organ or for a single instrument (41, 42) and there is no agreement on the gating strategy for essential immune subsets (41, 44). Furthermore, based on the experience of the authors, high dimensional panels such as these including 20-40 markers are difficult to customize and/or optimize. If there is a need to change a few markers to fit a particular study, it most likely requires substantial redesigning, hence limiting the scope of use of this type of panels in different studies. To address these issues and provide a practical and flexible but still reproducible and robust immune cell panel, we created a 13-marker backbone panel that identifies major immune cell subsets, and which can easily accommodate seven drop-in fluorochrome placeholders to allow researchers to add markers according to their specific study goals with minimal impact on the resolution of each immune cell population. With the incorporation of a Live/Dead fluorescent probe, this is a 14-parameter panel (13 markers + viability dye) expandable to at least 21 parameters without any need for redesign. Our panel is organ- and tumor-agnostic and uses standard tissue dissociation methods. The panel can also be employed to study complex TMEs such as that of pancreatic ductal adenocarcinoma and is compatible with analyzing bright fluorescent protein-expressing gene reporters, such as tdTomato, within the hematopoietic system. Finally, our backbone panel performance is consistent across all the major spectral flow cytometer systems currently available (i.e., Cytek Aurora, Sony ID 7000, BD FACSymphony S6 SE), making it a dependable and widely applicable tool for researchers to study immune cell populations in murine cancer models.

## 2 Materials and methods

### 2.1 Mice

Mice were maintained under specific pathogen-free conditions, in a controlled environment that maintained a 12-hour light-dark cycle, and food and water were provided *ad libitum*. The following mice were used: 6-10 weeks-old C57B6/N (purchased from Charles River) and C57BL/6J mice (purchased from the Jackson Laboratories), and in-house tdTomato^+^ *HSC-Scl-Cre-ER^T^* mice carrying *Tet2^flox/flox^* alleles (in a C57BL/6J background), and *Kras^G12C/+^;Trp53^fl/fl^* mice (in a C57B6/N background). To induce gene recombination in Cre-ERT^2^ mice, tamoxifen (100 mg/kg, MCE, HY-13757A), dissolved in corn oil (Sigma-Aldrich, C8267), was administered via oral gavage with a one-day drug holiday between dosing. Mice were randomly selected for each experiment. The veterinary staff provided regular monitoring and husbandry care, which included the appropriate housing, feeding, and cleaning of the animals. The mice were monitored daily for signs of disease or morbidity, such as bleeding, infection, fatigue, or failure to thrive, and any such signs were immediately addressed by sacrificing the animal. Additionally, they had intact immune systems and had not undergone any prior procedures. For the immunophenotyping comparison between wildtype and tdTomato^+^ *HSC-Scl-Cre-ER^T^ Tet2 ^flox/flox^* mice, each group consisted of 12-15 subjects, with a nearly equal distribution of male and female mice aged 30 weeks. C57B6/N female mice were specifically used for the generation of the syngeneic lung and pancreatic cancer models as described in the following sub-section (2.2).

### 2.2 Generation of syngeneic murine cancer models

For pancreatic cancer, pancreatic ductal epithelial cells (PDEC) that harbor an endogenous *Kras^G12D^* allele (45, 46) were electroporated with 1 μL Cas9-Cy3 (PNA Bio, CP06-100) and 1 μL 100 μM synthetic guide targeting *Trp53* (Synthego, ACCCTGTCACCGAGACCCC). Two days later, Cy3^+^ cells were sorted on an MA900 (Sony). The sorted cells were cultured in 10 μM Nutlin-3a (Selleck 1061) for 1 week. For orthotopic transplants of the *p53* knockout PDEC cells, mice were anesthetized, and a survival surgery was performed to expose the pancreas. 100,000 PDEC cells resuspended in 25 μL of cold 1:1 OptiMEM (Thermo Fisher, 31985062) and Matrigel (Corning, 354230) were injected into the tail region of the pancreas. Mice were monitored for tumor formation by abdominal palpation and euthanized after 5.5 weeks. For lung cancer models, 75,000 cells derived from a lung tumor formed in a C57B6 *^KrasG12C/+^;Trp53^fl/fl^* mouse were resuspended in 200 μL 1X PBS and injected into the tail vein of mice. Mice were assessed daily for distress signs, cachexia, weight loss over 20%, breathing difficulties, or tumors larger than 12 mm (no tumors surpassed this size limit) until 3.5 weeks post-transplant, when they were eventually euthanized. An age- and sex-matched group of mice were used as control (n=3-6) for all the cohorts.

### 2.3 Preparation of flow cytometry samples

To harvest the organs, mice were euthanized using CO_2_ asphyxiation. A submandibular bleed was performed to isolate peripheral blood, and 15 μL of whole blood was lysed with RBC lysis buffer (BioLegend, 420302), previously diluted to 1X with distilled water. To isolate the bone marrow, the femur, hip, and tibia were dissected and cleaned before being crushed on ice using a mortar. The harvested cells were spun down in FACS buffer (1X PBS + 2% FBS). After discarding the supernatant, pelleted cells were resuspended and incubated in 1X RBC lysis buffer. Spleens were mechanically disrupted with the back of a 5-mL syringe, filtered through a 70-μM strainer, washed with FACS buffer, and subsequently lysed with 1X RBC lysis buffer.

For the liver, the MACS liver dissociation kit (Miltenyi Biotec, 130-1-5-807) was used for dissociation according to the manufacturer’s protocol, using C tubes (Miltenyi Biotec, 130-096-334) and incubating on the gentle MACS OctoDissociator (program: 37°C m_LDK_1). The resulting cell suspension was filtered through a 70-μm strainer and washed with FACS buffer prior to red blood cell lysis with 1X RBC lysis buffer. Pancreata were cut into small 2- to 4-mm fragments in ice-cold FACS buffer. The fragments were then transferred to a solution of collagenase V (1 mg/ml, Sigma, C9263) for tumors or collagenase D (1 mg/ml, Roche, 11088882001) for normal pancreas with dispase II (2U/ml, Roche, 04942078001), soybean trypsin inhibitor (0.1mg/ml, Gibco, 17075029), and DNase I (0.1 mg/ml Roche, 04716728001) –all in 1X HBSS (Gibco, 14025076). The suspension was transferred to a GentleMACS C-tube and incubated on the OctoDissociator using the program “37°C m_TDK_1”. After incubation, cells were pelleted, resuspended in 0.05% Trypsin-EDTA (Gibco, 15400054), and incubated at 37°C for 5 minutes. Following the trypsin reaction, cells were spun down as above and washed in FACS buffer with DNase (0.1 mg/ml, Roche, 04716728001) and soybean trypsin inhibitor (0.1mg/ml, Gibco, 17075029). Red blood cell lysis was performed with 1X RBC lysis buffer. Cells were finally washed in PBS and resuspended in FACS buffer with DNase (0.1 mg/ml Roche, 04716728001) and soybean trypsin inhibitor (0.1mg/ml, Gibco, 17075029). Normal or cancerous lungs were first flushed with PBS. Next, they were dissociated with the MACS lung dissociation kit (Miltenyi Biotec, 130-095-927) according to the manufacturer’s protocol, using the program “37°C m_LDK_1” on the MACS OctoDissociator. After incubation, the cell pellet was filtered through a 70 μM cell strainer and spun down. The cell pellet was then resuspended in 1X RBC lysis buffer. For all samples, RBC lysis took 5 minutes on ice and was stopped by quenching with FACS buffer (at least doubling the amount of lysis buffer), and cells were subsequently spun down and resuspended in FACS buffer containing Fc Block. Every centrifugation or washing step was performed at 300 rcf for 5 minutes, at 4°C, and prior to Fc blocking, an incubation with Brilliant Stain Buffer (BD Horizon, 563794) took place at 4°C for 15 minutes, followed by a washing step.

To block the Fc receptors, we used the Purified Rat Anti-Mouse CD16/32 Fc Block (BD Biosciences, 553142, at a final dilution of 1:100) at 4°C for 10 minutes. We used fluorochrome-conjugated antibodies with the final concentrations specified in **Supplementary Table 1** of the **Supplementary Material**. This table also includes the antibodies’ manufacturer, catalog number, and purpose in this study. We determined the concentration of each antibody by titrating at least five dilutions per the saturation concentration. Using a sequential approach, we conducted the antibody staining in the dark at 4°C. First, we incubated the cells with the anti-CD3 antibody for 30-40 minutes, followed by incubation with the remaining antibodies for another 30-40 minutes, based on the panel used (i.e., backbone, immune, or TME panel, as indicated in **Supplementary Table 1**). We washed the cells with PBS and then incubated them with the Live/Dead Near-Infrared cell stain kit (Invitrogen, L10119) in the dark for 30 minutes at 4°C. Finally, we washed the samples twice with FACS buffer before resuspending the pellets in 200-300 μL of FACS buffer at a final concentration of 5,000-10,000 cells/μL. We stained between 1-2 million cells per sample in the same tube for all normal and tumor tissue specimens.

### 2.4 Flow cytometry single-stained controls

All single-stained controls were prepared using mouse splenocytes except for the drop-in controls where UltraComp eBeads ™ compensation beads (ThermoFisher Scientific, 01-2222-42) were used.

### 2.5 Flow cytometry acquisition on Cytek Aurora

Samples, including unstained and single-stained controls, were acquired on a five-laser Cytek Aurora spectral analyzer (355 nm, 405 nm, 488 nm, 561 nm, 640 nm) using Cytek Assay Settings (CAS) adjusted automatically for the 64 APD fluorescent detectors after running SpectroFlo® QC Beads (Cytek Biosciences, SKU B7-10001). Only forward- and side-scatter gains were manually adjusted to bring the events of interest in scale. After acquisition, unmixing using ordinary least squares (OLS) method was carefully performed with SpectroFlo® software, version 3.0.1. (Cytek Biosciences).

### 2.6 Flow cytometry acquisition on BD FACSymphony™ S6 SE

Samples, including unstained and single-stained controls, were acquired on a Spectrally Enabled (SE) five-laser BD FACSymphony™ S6 (355 nm, 405 nm, 488 nm, 561 nm, 637 nm) using optimal voltages determined by the manufacturer recommendation for each of the 48 PMT detectors, as described by Florian Mair and Aaron Tyznik (47). Only forward- and side-scatter gains were manually adjusted to bring the events of interest in scale. After acquisition, unmixing using OLS was carefully performed with BD FACSDiva™ software, version 9.6 (BD Biosciences).

### 2.7 Flow cytometry acquisition on Sony ID7000™

Unstained and single-stained controls were acquired on a 5-laser Sony ID7000™ spectral analyzer (355 nm, 405 nm, 488 nm, 561 nm, 637 nm) using optimal voltages adjusted automatically with QC Standardization mode for all 147 fluorescent PMT detectors. Only forward- and side-scatter gains were manually adjusted to bring the events of interest in scale. For fully stained samples, voltages were increased synchronously within each laser detection deck to the maximum while ensuring the signal in all channels was not saturated. After acquisition, unmixing using Weighted Least Square Method (WLSM) was performed with ID7000 system software, version 2.0.0.17121 (Sony Biotechnology).

### 2.8 Unmixing

Although there were different autofluorescence (AF) signatures for different organs, the AF of immune cells remained consistent. Therefore, for experiments involving non-fluorescent spleen, liver, bone marrow, and blood, we used unstained spleen cells as the reference spectral signature for AF. This same unmixing matrix was applied to all these samples. For pancreas tumor samples, we also employed the spleen AF signature, but we noted one population with a distinct signature compared to immune cells, exhibiting very high AF. To account for this, we exported the gated population from the unstained pancreas sample as an FCS file and reimported it as an extra parameter. The unmixing of pancreas tumor samples included both AF spectral signatures, and the same matrix was applied to both tumor and normal pancreas. For the lung tumor samples, we also included the same high AF in the unmixing and used the same unmixing matrix for both tumor and normal lung samples. For the wildtype and tdTomato^+^ *HSC-Scl-Cre-ER^T^ Tet2 ^flox/flox^* bone marrow samples, single-stained beads were used for all the markers, and unstained tdTomato^+^ *HSC-Scl-Cre-ER^T^ Tet2 ^flox/flox^* cells were used as the single-stained control for tdTomato. Non-fluorescent wildtype bone marrow cells were used as the unstained control.

### 2.9 Flow cytometry data analysis

Manual analysis was performed using FlowJo software, version 10.9.0, (BD Biosciences) and for unsupervised analysis with Omiq (Dotmatic) was used. Before analysis, data were cleaned by excluding debris, doublets, and dead cells (**Supplementary Figure S1**).

### 2.10 Analysis of sorted cells

Macrophages, monocytes, and neutrophils (20,000-50,000 of each cell population) were sorted and spun onto Cytospin slides after being resuspended in warm PBS at 350 g for 5 min. The slides were then air-dried overnight and stained using the Giemsa-Wright method. Pictures of the slides were taken using an Olympus BX53 bright microscope with an oil lens (x100) and x10 eyepiece, resulting in a total magnification of x1000.

### 2.11 Quantification, statistical analysis, and figure preparation

Data are presented as mean ± s.e.m. The statistical analysis was performed using two-way ANOVA with Geisser-Greenhouse correction to compare the population percentages across the three instruments (overall for instrument factor). The population percentages values were also compared between the two instruments (Aurora vs. ID700, Aurora vs. S6, ID700 vs. S6) using Tukey’s multiple comparisons tests. Two-tailed unpaired Student’s t-tests were used to compare the cell population percentages between WT and PDAC or WT and *Tet2^flox/flox^* samples, with the Welch’s correction being applied if the groups showed significantly different variances. Significance was set at p < 0.05, and statistical information can be found in the respective figure legends. GraphPad Prism 9 (GraphPad Software) was used to perform all statistical calculations. Figures were prepared using BioRender.com for scientific illustrations and Microsoft PowerPoint, version 16.54 (Microsoft) or Adobe Illustrator 2021 (Adobe) for the rest of figure panels.

## 3 Results

### 3.1 Backbone panel design and gating strategy

**Figure 1:**
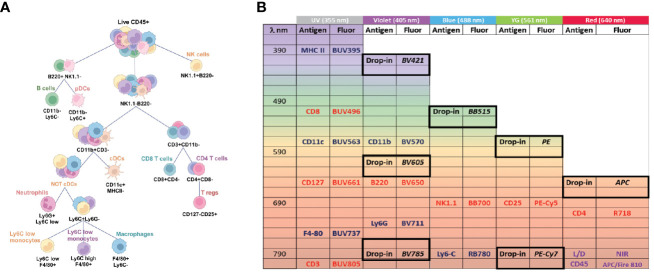
Backbone panel design and gating strategy. **(A)** Markers used to define the main immune populations as helper T cells, cytotoxic T cells, T regs, B cells, NK cells, pDCs, cDCs, macrophage, monocytes, and neutrophils. **(B)** Fluorochrome assignment. Drop-in positions are highlighted and suggested fluorochromes are written in italic. Myeloid markers are in blue, lymphoid markers in red. CD45, and Live/Dead dye are in purple.

To create the backbone panel, we developed a gating strategy to analyze the major lymphoid and myeloid populations, including T cells (CD4^+^ and CD8^+^ T cells), T regulatory cells (Tregs), Natural Killer (NK) cells, B cells, plasmacytoid dendritic cells (pDCs), conventional dendritic cells (cDCs), macrophages, monocytes (both Ly6C-low and high subsets), and neutrophils. We chose markers that can broadly define these immune cell populations (**Figure 1A**), while also leaving room for drop-in markers that could help us narrow down the subpopulations depending on the organ or analysis of interest (**Figure 1B**). For instance, if one wanted to characterize myeloid cells in the lungs, it would be necessary to add drop-in markers to distinguish between resident and recruited macrophages (48, 49).

Next, we carefully assigned the appropriate fluorochromes to each marker of interest, considering important principles of flow cytometry panel design, such as (I) aligning brightness of the fluorochrome with the level of antigen expression (i.e., brighter fluorochromes for lower expressed markers and vice-versa), and (II) minimizing emission overlap between fluorochromes conjugated to co-expressed markers to reduce spread (37, 50, 51). To ensure smooth performance of the backbone panel, we first selected fluorochromes for the drop-in positions to allow additional markers to be assigned without causing any disruption in panel resolution. We considered several important factors while choosing drop-in fluorochromes, including (I) minimal interference with the backbone fluorochromes and between each other, (II) effortless expansion (i.e., the drop-in fluorochromes must be minimally impacted by the backbone to allow for simple customization), (III) commercial availability of fluorochrome/antibody conjugates, and (IV) brightness.

Based on the criteria outlined above, we chose BV421, FITC or BB515, PE, and APC as our primary fluorochromes for drop-ins and evaluated BUV605, BUV786, and PE-Cy7 (tandem dyes) for their potential use as additional drop-ins. We opted for medium to high brightness fluorochromes for the drop-ins, as they are valuable for secondary or tertiary antigens (e.g., T cell activation and exhaustion markers). When assigning fluorochromes to the backbone markers, we prioritized minimizing the spectral overlap between co-expressed markers over brightness since most markers were primary antigens, and brightness was less of a concern. To minimize the spectral overlap, we alternated the allocation of lymphoid and myeloid markers across laser lines while also considering cross-laser excitation (**Figure 1B**). Given that CD45 was co-expressed with all other markers, we assigned the fluorochrome APC-Fire 810 with a unique spectral signature to minimize interference with the remaining markers. We then chose the near infrared Live/Dead viability dye with high similarity to APC-Fire 810 to enable us to select the single positive population for live CD45^+^ cells without the cost of an additional unique dye. To label MHC II expressed by both B cells and various myeloid subtypes, we designated BUV395, a dim dye, to minimize any impact on the other markers. Similarly, we assigned BUV496 to the highly expressed CD8 molecule and BUV563 to CD11c, which is well expressed in DCs, both of which are also dim fluorochromes.

To reduce the spreading effect, we paired BUV661, a moderately bright dye with some potential emission overlap with APC (reserved for a drop-in marker), with CD127, a marker expressed by T cells at low levels. For F4/80, a macrophage marker, we chose the moderately bright fluorochrome BUV737. To avoid any spread on all the T cell markers, we selected BUV805 for CD3 due to its unique spectrum emission and low overlap with other fluorochromes. For CD4, we chose R718, a dye excited by the red laser with minimum spread on the drop-in reserved for APC. We also carefully considered fluorochromes that may introduce or be susceptible to excessive spectral spreading, with markers expressed by cell types less likely to have added drop-ins, or due to their lack of subtypes or co-expression with other backbone markers. For example, we chose BV711 (a potentially problematic dye) for Ly6G, which is expressed only by neutrophils, and similarly BV650 for B220, which is expressed by B cells and pDCs. Finally, instead of using Foxp-3 as the Treg primary marker, we strategically opted for gating Tregs as CD25^+^, CD127^-^ cells. This allowed compatibility with fluorescent protein-expressing murine models whose fluorescence signal may be impacted by permeabilization and fixation protocols (52). With this approach, we were able to design a meticulous antibody panel for robust and accurate flow cytometry analysis (**Supplementary Figure S2**).

### 3.2 Evaluation of the backbone panel and impact on drop-in channels

**Figure 2:**
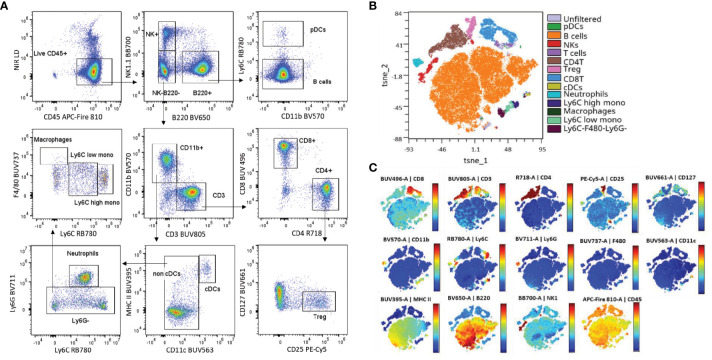
The backbone panel efficiently resolves the main murine immune populations. **(A)** Manual gating strategy applied to spleen cells stained with the backbone panel. **(B)** t-SNE scatter plot overlayed with the manual gated populations (t-SNE iterations = 1000 and k = 30). **(C)** Colored t-SNE scatterplots showing the expression level and distribution of the backbone markers.

To evaluate the performance of the backbone panel, we first verified the accuracy of the single-stained controls by visualizing the N x N plots (**Supplementary Figure S3**). Our evaluation involved a manual gating approach, which enabled us to successfully identify the key target populations. **Figure 2A** depicts representative plots using splenocytes from wildtype (WT) C57BL/6J mice. In addition, we utilized the T-distributed stochastic neighbor embedding (T-SNE) dimensionality reduction algorithm and overlayed manual gating to the resulting plots. This unsupervised analysis further confirmed the effectiveness of our fluorochrome selection in identifying different immune cell populations (**Figures 2B, C**).

**Figure 3:**
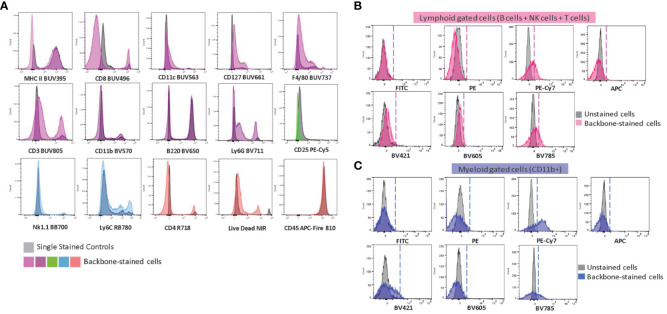
Evaluation of the backbone panel and impact on drop-in fluorochromes. **(A)** Histograms of single-stained spleen cells overlaid with backbone-stained spleen cells. Single-stained samples for each fluorochrome are in grey. A representative sample stained with the complete backbone panel is colored. **(B)** Histograms showing the impact of the backbone on the drop-in fluorochromes (FITC, PE, PE-Cy7, APC, BV421, BV605 and BV785). Unstained splenocytes (in gray) overlaid with the backbone-stained splenocytes gated on the lymphoid cells (T, B and NK cells –in pink). **(C)** Unstained splenocytes (in gray) overlaid with the backbone-stained splenocytes gated on the myeloid cells (CD11b+ cells –in blue).

We then conducted a comprehensive analysis of the samples that were stained with the complete backbone antibody cocktail, in addition to those stained only with each individual antibody. When we added all the antibodies of the backbone panel together, there was no impact on the brightness of the positive signal (**Figure 3A**). Although there was spreading observed in the fluorochromes of certain myeloid markers, such as CD11c (BUV563), F4/80 (BUV737), and Ly6G (BV711), the distinctively high expression of such markers ensured that the resolution of the cell populations remained unaffected. It is worth mentioning that we specifically chose these fluorochromes to avoid any interference with the drop-ins, which operate at shorter wavelengths and are well separated from the far-red range. As for lymphoid markers, we observed a slight reduction in the negative signal of CD3 (BUV805) due to spreading. To achieve a higher resolution of the CD3^+^ population, we had to extend the incubation period with the anti-CD3 antibody. This involved a preliminary step where we stained the sample with the anti-CD3 antibody for 30 minutes, followed by the addition of the remaining antibodies and a 30-minute incubation (**Supplementary Figure S4**). This observation emphasizes the importance of sequential incubation or a longer incubation time to maintain signal intensity and resolution for a specific antibody, as other authors have similarly reported (50, 53).

We also evaluated the impact of the backbone panel on the signal from the drop-in fluorochromes. For this purpose, we generated an unmixing matrix using cells that were single-stained with different anti-CD4 antibodies conjugated with seven drop-in fluorochromes (i.e., FITC, PE, PE-Cy7, APC, BV421, BV605, BV785). The overlap between unstained cells and those stained with the backbone panel provided insights into the signal from drop-in fluorochromes separately for lymphoid and myeloid cells. This analysis validated our selection of drop-ins and helped us assess the impact of fluorochrome choices on distinct populations. We found that the lymphoid population had a minimal impact in reducing the resolution of the drop-in fluorochromes (**Figure 3B**). In contrast, the myeloid population had a more significant effect on PE-Cy7 and BV785 (**Figure 3C**). This was not surprising because PE-Cy7 has a similar emission spectrum as RB780 (conjugated to Ly6C) and BV785 is akin to BV711 (conjugated to Ly6G). In general, fluorochrome signatures with higher similarity, i.e., a higher emission spectrum overlap, tend to cause spreading errors (47). This finding implied that these fluorochromes may not be suitable in combination with co-expressed markers on myeloid cells. One should avoid PE-Cy7 for neutrophil markers and BV785 for Ly6C-expressing cells. We, therefore, decided to use these drop-in channels for lymphoid co-expression markers or makers of non-immune cell types such as tumor stromal cells or cancer cells in solid tumors.

### 3.3 The performance of the backbone panel is reproducible across different spectral flow cytometers

As an attempt to evaluate the consistency of our backbone panel, we conducted an experiment to assess its reproducibility in different spectral flow cytometers equipped with the same laser lines but distinct detection platforms. Specifically, we assessed three instruments –Cytek Aurora, Sony ID7000, and BD FACSymphony S6 SE– and analyzed the same sample source (splenocytes isolated from three WT C57BL/6J mice) after staining with the backbone panel. We employed the same single-stained controls to calculate unmixing matrices for each cytometer to ensure consistency and accuracy.

**Figure 4:**
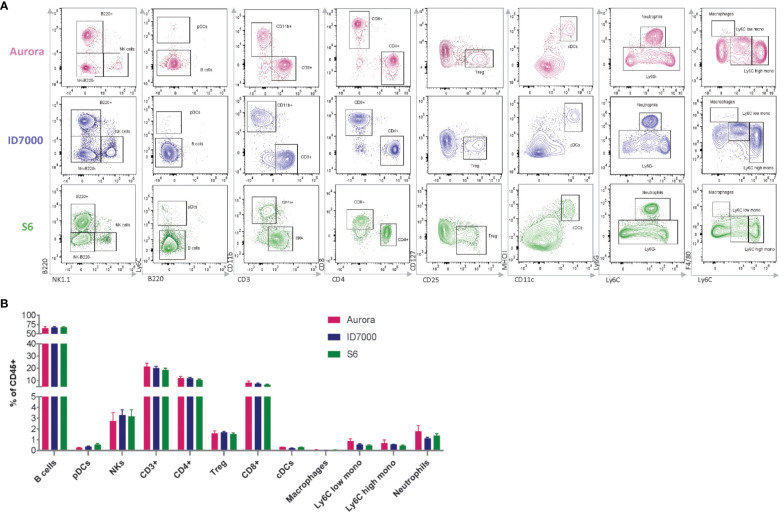
The performance of the backbone is reproducible across different spectral flow cytometers. **(A)** Manual gating strategy showing the main immune populations. Wildtype spleen cells were stained with the backbone panel and acquired on Cytek Aurora (in pink –top), Sony ID 7000 (in blue –middle), and BD S6 SE (in green –bottom). **(B)** Comparison of the frequency of the main immune populations of live CD45^+^ cells shows no significant differences across the three instruments (n=3 mice/instrument). Data are ± mean s.e.m. Statistical analysis was performed using two-way ANOVA with Geisser-Greenhouse correction, followed by Tukey’s multiple comparisons tests to compare the mean values of the immune cell population percentages between the two instruments (all p values were > 0.1.).

Though the results showed some variations in signal intensity among the instruments, with Cytek Aurora showing the highest intensity and BD FACSymphony S6 SE the lowest, our manual gating approach effectively identified the primary immune cell populations with minimal variation (**Figure 4A**). This finding underscored the robustness of our backbone panel and its potential use in various spectral flow cytometry systems. To further assess the backbone’s reliability across different spectral platforms, we compared the population frequencies across the three instruments and found no statistically significant differences among the three devices (p = 0.3119) (**Figure 4B**). This encouraging outcome demonstrated that our backbone panel is a powerful and dependable tool for researchers conducting studies across different spectral flow cytometry systems. Additionally, we tested the backbone panel for sorting different immune cell populations and successfully sorted neutrophils, macrophages, and monocytes (**Supplementary Figure S5**) that can be used for downstream applications, from cell culture to genomic analyses.

### 3.4 Impact of drop-ins on the backbone-defined immune populations

**Figure 5:**
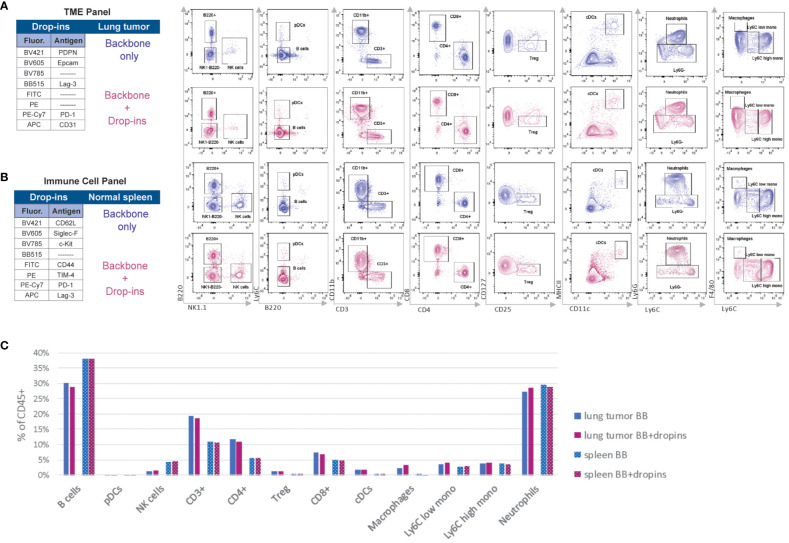
The backbone-defined immune populations are unaffected by the addition of drop-in markers. **(A)** Manual gating strategy applied to cells harvested from *Kras^G12C/+^; Trp53^fl/fl^* lung adenocarcinoma derived from a syngeneic mouse model stained with the backbone panel only and backbone plus drop-ins of the tumor microenvironment (TME) panel (i.e., Epcam, CD31, PDPN, PD-1, and Lag-3). **(B)** Manual gating strategy applied to wildtype spleen cells stained with the backbone panel only and backbone plus drop-ins of the immune cell panel (i.e., CD62L Siglec-F, c-Kit, CD44, TIM-4, PD-1, and Lag-3). **(C)** Comparison of the frequency of the backbone-defined immune population within live CD45^+^ cells in the presence or absence of drop-ins in lung tumor and spleen samples.

Next, we wanted to ensure the adaptability and resolution of our backbone panel for specific biological contexts, which involved incorporating two separate drop-in panels: (I) the immune cell panel and (II) the TME panel. We specifically designed these panels to study immune checkpoints and stromal cells in the TME, or simply expand the number of immune cell markers, thereby enabling the detection of eosinophils, memory/effector T cells, c-Kit expressing cells (i.e., cancer cells/blasts when examining the peripheral blood, or hematopoietic stem/progenitor cells (HSPCs) in the bone marrow/spleen), and immune checkpoint markers (**Figure 5A**).

For each panel, we used different samples. While we utilized spleen cells pooled from WT C57BL/6J mice for the immune cell panel (**Supplementary Figure S6**), we analyzed a pooled single-cell suspension of *KRAS*-driven lung adenocarcinoma for the TME panel (**Supplementary Figure S7**). For both panels, we compared samples stained with the backbone panel to those co-stained with the backbone panel plus relevant drop-in markers (**Figures 5A, B**). We found no discernible differences in signal resolution or frequency of the backbone immune cell populations between these two groups of samples (**Figure 5C**). This result indicated that the drop-in fluorochromes had no negative impact on the performance of the backbone panel. Thus, the backbone design is highly versatile and adaptable, making it well-suited for complex immunophenotyping studies.

### 3.5 The backbone panel is organ-agnostic and allows for comparison of immune cell populations across different tissue types

Although markers of immune cell subtypes are the same across different tissues, including hematopoietic and non-hematopoietic organs, distribution patterns of immune populations differ significantly depending on the site and the pathological context (54, 55). Thus, we sought to prove that the backbone panel could efficiently resolve the immune cell populations in different tissues. We processed samples from a WT C57BL/6J mouse’s spleen, blood, bone marrow, liver, and lung. We stained all samples with the backbone antibody cocktail and analyzed them on the Cytek Aurora using the same parameters.

**Figure 6:**
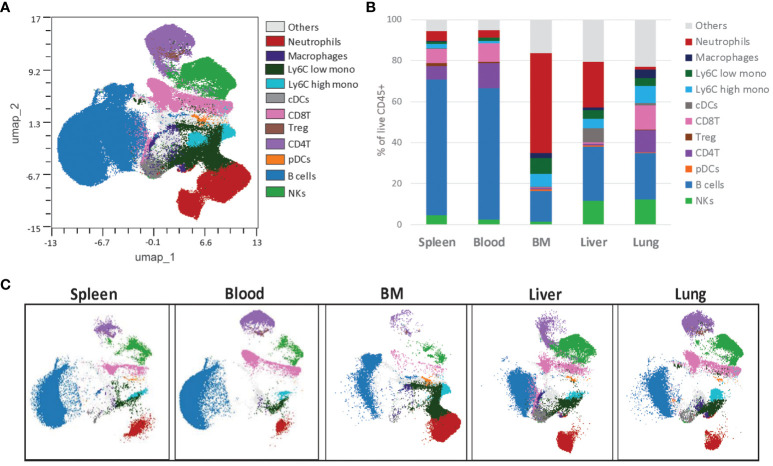
The backbone panel is organ-agnostic. **(A)** The UMAP scatter plot shows concatenated events from six different organs, and the overlay shows the distribution of the manual gated cells. For the UMAP analysis, each organ sample was downsized to 40,000 of manual gated live CD45^+^ singlet cells. **(B)** Individual UMAP scatterplot showing the differences between the organs, namely spleen, blood, bone marrow (BM), liver and lung. **(C)** Distribution of the immune populations frequency within live CD45^+^ cells, manually gated.

Despite different organs having varying expression levels and distributions for various immune cell markers, we could consolidate the data from all into a single uniform manifold approximation and projection for dimension reduction (UMAP) map (**Figure 6A**). Furthermore, we created independent UMAP plots for each tissue type. All the detected immune cell populations from the concatenated UMAP were represented in each sample with tissue-specific densities (**Figure 6B**). This proves the backbone panel’s suitability for analyzing immune cell populations in major organs and allows for percentual comparisons of each immune cell population across tissues, as we indicated here (**Figure 6C**). The backbone panel’s ability to detect all immune cell types across various tissue types is a significant advancement in our field.

### 3.6 Scalability of the backbone panel is effective to study a complex tumor immune microenvironment

**Figure 7:**
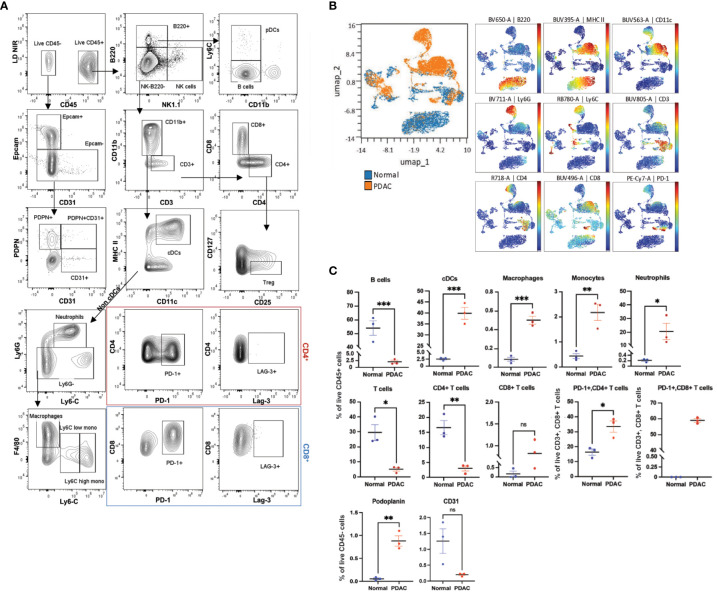
The backbone panel is efficient in analyzing the complex pancreatic ductal adenocarcinoma microenvironment. **(A)** Manual analysis of cells harvested from pancreatic ductal adenocarcinoma (PDAC), pancreata stained with the backbone and the drop-ins of the TME panel (i.e., Epcam, CD31, PDPN, PD-1, and Lag-3). **(B)** UMAP scatter plot shows concatenated events from the six samples (three normal and three PDAC pancreata, n=3 mice/group). The overlay shows the distribution of normal and tumor cells. For the UMAP analysis, each sample was downsized to 10,000 manually gated from the live CD45+ singlet cells. The colored scatterplot shows the expression level and distribution of the markers with the most relevant differences. **(C)** Comparison of the manually gated populations between normal and PDAC samples. Bar graphs showing percentages of different cell populations. Data are ± mean s.e.m.; p values from two-tailed unpaired Student’s t-test (ns, non-significant; *p < 0.05, **p < 0.01, ***p < 0.001, ****p< 0.0001).

Once we confirmed the efficacy of the backbone panel in exploring the immune cell populations within different mouse tissues, we assessed its capability in investigating immune cell populations within the TME of pancreatic ductal adenocarcinoma (PDAC). PDAC is known for its intricate immune microenvironment (56). To examine the ability of the backbone panel in profiling the PDAC immune landscape, we utilized syngeneic models implanted with *Kras^G12D/+^*; *Trp53^Cas9-KO^* pancreatic ductal epithelial cells (PDEC) (45, 46). In total, we profiled 1.5 million cells with an average of 300,000 events per sample using the TME panel. We implemented a thorough gating strategy, as shown in **Figure 7A**, to identify relevant PDAC cell populations, including (I) immune cell populations and checkpoint markers, (II) epithelial cells (Ep-CAM^+^), (III) endothelial cells (CD31^+^), (IV) fibroblastic reticular cells (Podoplanin^+^ (PDPN)), and (V) lymphatic endothelial cells (CD31 and PDPN-double positive cells). Unsupervised UMAP analysis showed differences in the immune cell distribution between normal (WT) and PDAC pancreata when concatenating and clustering different samples together, allowing us to identify major immune cell populations and separation between normal and PDAC samples (**Figure 7B**).

In the PDAC pancreata, we observed a predominant myeloid cell infiltration, including monocytes, macrophages, neutrophils, and cDCs, and a concomitant significant decrease in the proportion of B and T cells in comparison to the normal tissue, which reflected a deficient adaptive immune cell response. We also found that CD8^+^ T cells showed an increasing trend in PDAC, although two-thirds of these expressed the exhaustion marker PD-1 (**Figure 7C**). Furthermore, PDAC samples contained higher levels of PDPN^+^ but a decreasing trend in the percentage of CD31^+^ cells, although the latter was not statistically significant (**Figure 7C**). These results aligned with previous studies characterizing immune cells and TME in PDAC (57–60) and confirmed the adaptability and effectiveness of our backbone panel in studying cancer types with complex immune cell microenvironments.

### 3.7 The backbone can be used in combination with a bright fluorescent protein and drop-ins

When conducting flow cytometry, high fluorescence levels, such as that from a fluorescent protein, can pose a significant challenge as it may spread and impact signal resolution. This is especially true for fluorescent proteins that have a broad emission spectrum and can overlap with many fluorochromes (52, 61). Therefore, it was essential to test the efficacy of the backbone panel in combination with a bright, strongly expressed fluorescent reporter to pinpoint immune differences accurately, as many genetically engineered mouse cancer models express fluorescent gene reporters.

For this evaluation, we used C57BL/6J mice with the hematopoietic stem cell (HSC)-specific, tamoxifen-inducible Cre recombinase (*HSC-Scl-Cre-ER^T^*) and a Cre-inducible tdTomato (tdT) reporter, which efficiently and specifically targets adult hematopoietic cells at the stem/progenitor cell level, rendering them tdT^+^ (62). These mice have been extensively used in hematopoietic fate-cell tracing studies and are now being utilized to study clonal hematopoiesis (CH) and leukemia (63–68). Specifically, we sought to characterize *Tet2* loss in these models. Loss-of-function somatic mutations in *TET2* are associated with various types of hematopoietic cancers in humans, including myeloid and lymphoid cancers as well as several solid cancers (69). These mutations are also often observed in preleukemia conditions such as CH, which is the expansion of hematopoietic stem cell clones related to age (70). As one of the most prevalent mutations affecting hematopoiesis, several research groups –including the Levine Lab– have established murine models of *Tet2* loss (64, 71–73).

**Figure 8:**
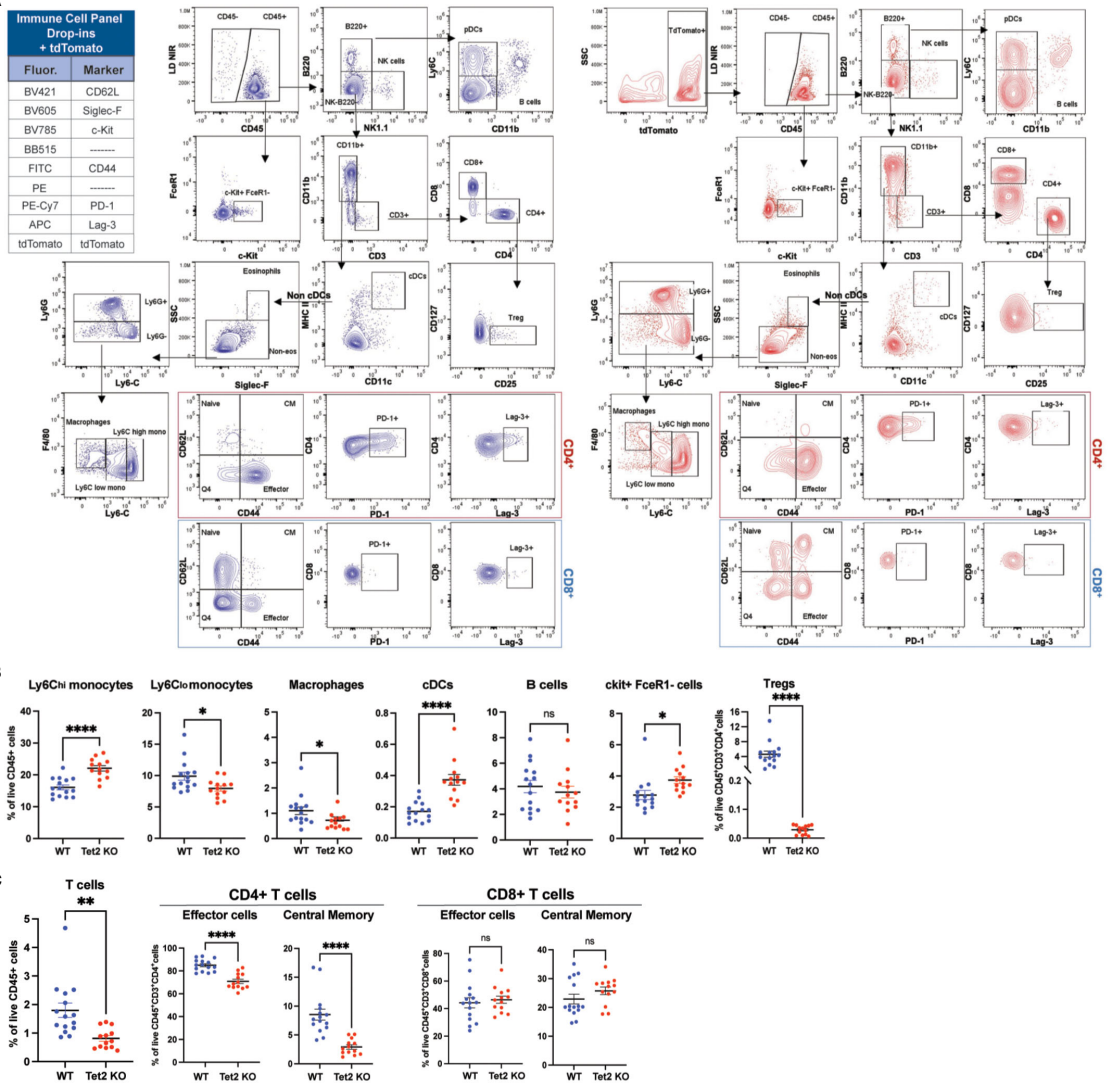
The backbone panel efficiently works in the presence of a tdTomato fluorescent and other drop-in fluorochromes and allows for an immune cell characterization of tdTomato *Tet2^KO^* mice relative to WT. **(A)** Manual analysis of cells harvested from whole bone marrow of WT (non-tdT) and tdT-expressing *Tet2^KO^* mice (tdT). To immunophenotype *Tet2* loss in *HSC-Scl-Cre-ER^T^* mice, we previously crossed them to *Tet2^flox/flox^* to make a phenotyping comparison between Cre^+^ (*Tet2^Knockout (KO)^*) mice –expressing tdT– and their age-matched Cre^-^ (functionally WT mice) counterparts –lacking tdT. **(B)** Comparison of the manually gated bone marrow immune cell populations between WT and *Tet2^KO^* mice. Bar graphs showing percentages of myeloid and lymphoid cell populations as percentage of live CD45^+^ cells. Data are ± mean s.e.m.; p values from two-tailed unpaired Student’s t-test (ns, non-significant; *p < 0.05, **p < 0.01, ***p < 0.001, ****p< 0.0001). **(C)** Comparison of effector and memory CD4^+^ and CD8^+^ T cells in the bone marrow between WT and *Tet2^KO^* mice. Data are ± mean s.e.m.; p values from two-tailed unpaired Student’s t-test (ns, non-significant; *p < 0.05, **p < 0.01, ***p < 0.001, ****p< 0.0001).

To immunophenotype *Tet2* loss in *HSC-Scl-Cre-ER^T^* mice, we previously crossed them to *Tet2^flox/flox^* to make a phenotyping comparison between Cre^+^ (*Tet2^Knockout (KO)^*) mice –expressing tdT– and their age-matched Cre^-^ (functionally WT mice) counterparts –lacking tdT. We isolated whole bone marrow and stained with the backbone cocktail antibodies in addition to antibodies for drop-in markers, including Siglec-F for eosinophils, c-Kit for HSCPs, and CD62L, CD44, PD-1, and Lag-3 for T cell activation, and exhaustion. In total, we profiled 1.5 million cells with an average of 100,000 events per sample. Despite the high tdT brightness (10^5^-10^6^), the ability of the backbone panel to identify the different immune cell populations remained unaffected and we were able to detect differences in specific immune populations between the two mouse groups (**Figure 8A**). *Tet2^KO^* mice showed an overall increased frequency of myeloid cells relative to WT, with an increase in the percentage of proinflammatory Ly6C-high monocytes but reduced percentages of Ly6C-low monocytes and macrophages, indicating elevated inflammation at steady state. Additionally, the cDC population was increased in *Tet2^KO^* mice, which probably differentiated from Ly6C-high monocytes and was proinflammatory (**Figure 8B**). Regarding the lymphoid compartment, we detected a significant reduction in the overall CD3^+^ T cell population and Tregs in *Tet2^KO^* mice, as well as a reduction in both effector and central memory CD4^+^ T cells, indicating impaired differentiation of *Tet2^KO^* CD4^+^ T cells (**Figure 8B**). The percentages of naïve CD4^+^ and CD8^+^ T cells, the expression of exhaustion markers, and percentages of effector/memory CD8^+^ T cells did not change significantly (**Figures 8A, C**). However, the percentage of B cells showed a trend towards a reduction in *Tet2^KO^* mice (**Figure 8B**), suggesting there are pleiotropic effects of *Tet2* loss in the lymphoid lineage. Finally, by adding the c-kit marker, we could compare total HSPC percentages and found that the bone marrow of *Tet2^KO^* mice had a significantly higher percentage of CD45^+^c-kit^+^ cells (**Figure 8B**), after excluding mast cells (FcϵR1^+^, c-kit^+^) (**Figure 8A**), which suggested an increase in HSPC self-renewal *in vivo*. This practical example showcases the ability of our backbone panel to operate at a high resolution in the presence of a fluorescent reporter, offering great power and capability for experiments.

## 4 Discussion

In immune profiling studies, developing an effective flow cytometry panel is essential to obtaining reproducibility and avoidance of artifacts. However, designing and validating a high-dimensional flow cytometry panel can be extremely challenging as it requires not only expert knowledge of the biological markers required to define the cellular populations to be interrogated, but also significant technical expertise in flow cytometry and in the principles of panel design and validation. Here, we aimed to design and evaluate a versatile backbone panel for spectral flow cytometry, which allows for robust and customizable immune cell analysis across various tissues and immune microenvironments in mice. While there are already many proposed panels for the profiling of murine immune cells using more than 13 markers (38–41, 74, 75), the combinatorial nature of these high-parameter panels and all the complex rules that need to be adhered can make it as challenging to modify only a few parameters to adapt to the research question as it is to build an entirely new panel. These panels were designed as a whole, and most changes can have a profound effect on the overall panel resolution. Further, most of the current panels were designed for a specific polychromatic flow cytometry experiment, and only a few were tested on a single spectral flow cytometry platform (38–41). To our knowledge, ours is the first murine backbone panel validated across different spectral instruments; thus, this panel is a valuable resource for researchers who have access to any of the current spectral flow cytometer systems.

Our backbone panel includes the most common markers used to define immune cell populations and we do not propose a new gating strategy or marker combination to define immune populations. Instead, the backbone panel is a rigorously validated tool for scientists to expand upon to best suit their research questions and optimized to minimize the impact on relevant fluorochromes by comparing fully stained backbone cells with unstained samples. Noteworthy, the drop-in positions can be filled by similar fluorochromes beyond those suggested, whether they are commercially available or purchased through custom conjugations offered by different reagent companies. Furthermore, the number of drop-in positions can be expanded using some of the emission gaps we indicated in the fluorochrome assignment chart (**Figure 1B**), and their impact on the backbone panel and vice-versa can be validated using the same approach presented here. For the backbone markers, we were not concerned about fluorochrome brightness because most of the lineage markers were highly expressed. Further, we did not select common fluorochromes since these markers had enough commercial options readily available. Instead, we considered the similarity and possible spreading among them. To overcome this, we intercalated lymphoid markers with myeloid markers on the same laser line while also considering cross-laser excitation (**Figure 1B**). We strategically selected the most unique fluorochromes for markers present in many subtypes of cells (e.g., CD45, CD3, MHCII) and those fluorochromes that were more likely to cause spread and impact the resolution of others to markers expressed by a sole cell population or distinct population (e.g., Ly6G and B220) (**Supplementary Figure S2**). Our assessment of the backbone panel for spectral flow cytometry demonstrated its efficacy in the analysis of immune cell populations across various tissue types. This feature enables the comparison of specific immune cell populations in different tissues to assess organ infiltration, metastasis, and residual disease despite distinct organ-specific characteristics. Although our results already show a consistent identification of the expected immune populations in different tissues (**Figure 6**), these variations can be further minimized by exploring the presence of different autofluorescence signatures within the same tissue to improve resolution. Once these different signatures are identified, the use of autofluorescence extraction tools can remove the noise introduced by cellular autofluorescence and improve separation between negative and positive populations.

We demonstrated the ability of the backbone to profile the immune contexture of complex TMEs, as shown in our practical application of the PDAC immune profiling. We used a syngeneic *Kras^G12D/+^; Trp53^Cas9-KO^* mouse model and were able to detect statistically significant changes in PDAC, such as an increase in the levels of myeloid cells (i.e., cDCs, neutrophils, macrophages, and monocytes) and a decrease in the proportion of B and T cells compared to normal pancreatic tissue (**Figures 7B, C**). This is consistent with previous immunophenotyping studies of advanced PDAC stages (56, 57). Furthermore, we identified stromal cells using drop-in markers (**Figure 7A**) and found a rise in the total percentage of PDPN^+^ cells (**Figure 7C**), with or without co-expression of CD31 (**Figure 7A**), indicating active fibroblast expansion and lymphangiogenesis (76). Although it was not statically significant, we also observed a decrease in the percentage of endothelial cells (**Figure 7A**), which was expected since PDAC is known to be a poorly vascularized tumor, which has been reported to be due to blood vessels being destroyed by cancer cell infiltration (77). Importantly, we found that the backbone panel is compatible with transgenic mouse cells expressing fluorescent proteins such as tdT. Here, we characterized the bone marrow immune environment of tdT^+^ *HSC-Scl-Cre-ER^T^ Tet2^flox/flox^* mice (62). We compared them to age-matched WT control mice lacking tdT expression (**Figure 8A**). *Tet2^KO^* mice had a proinflammatory, myeloid-biased phenotype, predominantly shown by an increased percentage Ly6C-high monocytes (**Figure 8B**). Remarkably, the percentage of cDCs was also significantly elevated (**Figure 8B**), supporting the idea that cDCs can contribute to *Tet2*-driven inflammation (78). However, we also observed defects in the lymphoid lineage, such as a significant Treg deficiency (**Figure 8B**) and a reduction in the percentages of effector and central memory CD4^+^ T cells; however undetected for CD8^+^ T cells (**Figure 8C**). These findings highlight the deleterious effects of *Tet2* loss in both myeloid and lymphoid cells, particularly in effector/memory CD4^+^ T cells and Tregs, ultimately impacting both innate and adaptive immune responses. These findings are consistent with previous reports, although some have utilized other *Tet2^KO^* models with deletion within specific hematopoietic cell subset(s), rather than HSPCs (64, 79). This suggests that *Tet2^KO^* defects are passed on to the progeny, which was previously reported for myeloid cells (64, 67, 80) but has yet to be better explored within the different lymphoid compartments. Additionally, the higher percentage of CD45^+^c-kit^+^, FcϵR1^-^ cells (**Figure 8B**) is consistent with the well-documented increase of *Tet2^KO^* HSPC self-renewal (64, 72, 73). The inclusion of c-kit^+^ in this panel (**Figure 8A**) also allows for the detection of blasts in the peripheral blood to assess leukemia progression –a percentage that should be nonexistent or negligible in the peripheral blood of WT mice. The changes we report here are consistent across mice within each group (*Tet2^KO^* and WT) and showcase the maintenance of the resolution of the backbone panel even in the presence of tdT in combination with drop-in markers, making it ideal for transgenic mouse research that incorporates fluorescent proteins.

In summary, our validated murine backbone panel for spectral flow cytometry is exceptionally robust yet adaptable and offers researchers significant benefits in immune cell profiling across different tissues, immune microenvironments, and experimental setups. Subsequent studies will assess the compatibility of the current backbone panel with intracellular markers, expand the number of drop-ins (e.g., to include both lineage-specific and HSPC makers), and adapt the backbone panel to other species to increase robustness and adaptability. We believe that with this approach, high-throughput analysis of immune cells *in vivo* will become more efficient and facilitate greater integration of datasets that will inform our understanding of the interplay between the immune system, cancer cells, and the heterogeneity of different hematologic subsets in the spectrum of disease states.

## Data availability statement

The raw data supporting the conclusions of this article will be made available by the authors, without undue reservation.

## Ethics statement

The animal study was approved by Memorial Sloan Kettering Cancer Center (MSKCC) under the Institutional Animal Care and Use Committee-approved animal protocols (#07-10-016 and #11-06-011). The Guide for the Care and Use of Laboratory Animals (National Academy of Sciences 1996) was also followed to guarantee that the animals were treated ethically and humanely. The study was conducted in accordance with the local legislation and institutional requirements.

## Author contributions

AL: Conceptualization, Data curation, Formal analysis, Investigation, Methodology, Software, Supervision, Writing – original draft, Writing – review & editing. IF-M: Conceptualization, Data curation, Formal analysis, Investigation, Methodology, Software, Supervision, Writing – original draft, Writing – review & editing. MK: Investigation, Methodology, Writing – review & editing. MW: Investigation, Methodology, Writing – review & editing. SM: Methodology, Writing – review & editing. WX: Methodology, Writing – review & editing. SL: Supervision, Writing – review & editing. RL: Conceptualization, Funding acquisition, Supervision, Visualization, Writing – review & editing. RG: Conceptualization, Resources, Supervision, Writing – original draft, Writing – review & editing.
