# Supplementary material for: Development of a customizable mouse backbone spectral flow cytometry panel to delineate immune cell populations in normal and tumor tissues
Source: Front Immunol. 2024 Mar 27;15:1374943. doi: 10.3389/fimmu.2024.1374943 (PMC11008467; doi:10.3389/fimmu.2024.1374943)
Supplement: Supplementary file 1 [file DataSheet_1.pdf]

Supplementary Material

Development of a customizable mouse backbone spectral flow cytometry panel to delineate immune cell populations in normal and tumor tissues.

Ana Leda F. Longhini[*,†,1], Inés Fernández-Maestre[†,2,3], Margaret C. Kennedy[3,4], Matthew G. Wereski[2], Shoron Mowla[2], Wenbin Xiao[2,5,6], Scott W. Lowe[4,7], Ross L. Levine[*,†,2,5,8], Rui Gardner[*,†,1]

[1]Flow Cytometry Core Facility, Memorial Sloan Kettering Cancer Center (MSKCC), New York, NY, USA
[2]Human Oncology and Pathogenesis Program, Memorial Sloan Kettering Cancer Center, New York, NY, USA
[3]Louis V. Gerstner Jr Graduate School of Biomedical Sciences, Memorial Sloan Kettering Cancer Center, New York, NY, USA
[4]Department of Cancer Biology and Genetics, Memorial Sloan Kettering Cancer Center, New York, NY, USA
[5]Center for Hematologic Malignancies, Memorial Sloan Kettering Cancer Center, New York, NY, USA
[6]Department of Pathology and Laboratory Medicine, Hematopathology Service, Memorial Sloan Kettering Cancer Center, New York, NY, USA
[7]Howard Hughes Medical Institute, Memorial Sloan Kettering Cancer Center, USA
[8]Department of Medicine, Leukemia Service, Memorial Sloan Kettering Cancer Center, New York, NY, USA

[†]These authors contributed equally: Ana Leda F. Longhini, Inés Fernández-Maestre, Ross L. Levine, Rui Gardner

*** Correspondence:**
Dr. Ana Leda F. Longhini (figueia@mskcc.org)

Dr. Ross L. Levine (leviner@mskcc.org)

Dr. Rui Gardner (gardnerr@mskcc.org)

Keywords: Tumor Microenvironment (TME), Backbone Panel, Immune Cells, Spectral Flow Cytometry, Mouse, Immunophenotyping.

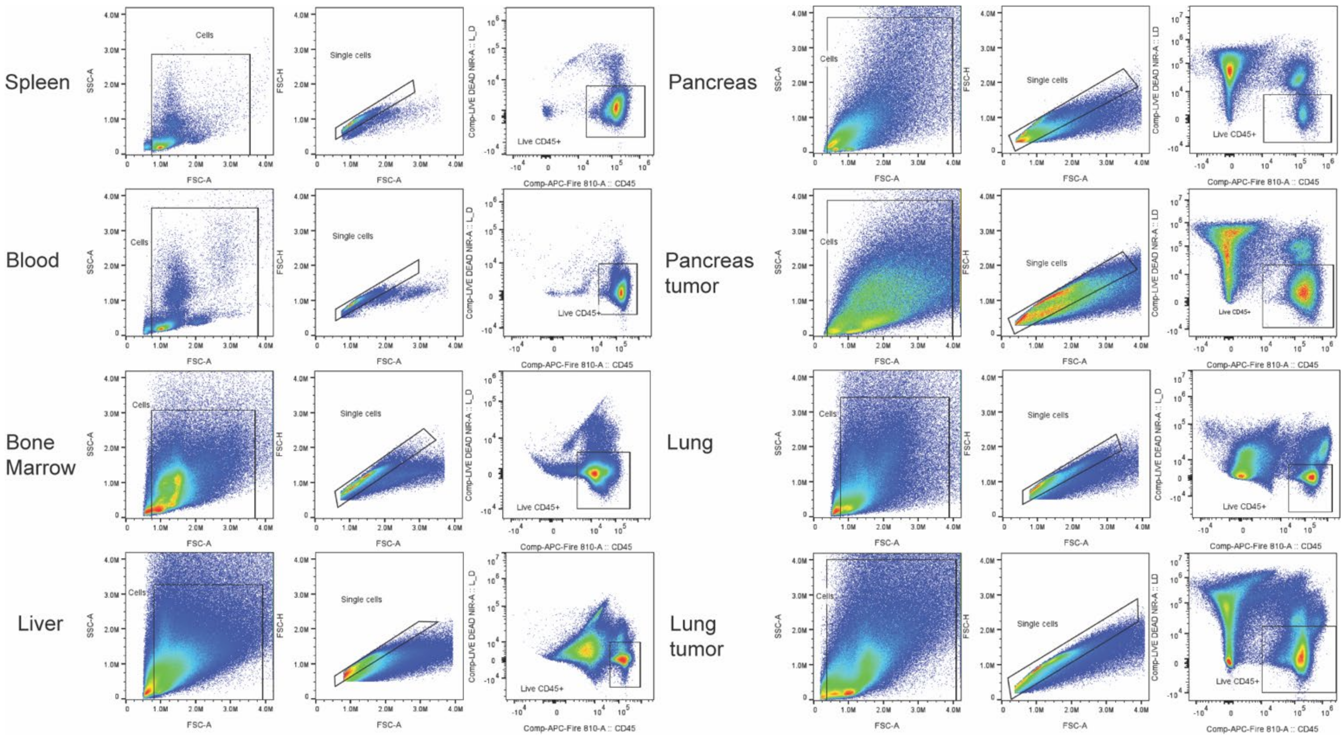

Supplementary Figure S1. Data cleaning examples for different sample types prior to the backbone gating strategy. Representative plots of various organs acquired on Cytek Aurora are depicted.

Laser	Fluorochrome	Marker	Rationale
UV	BUV395	MHC II	Medium brightness - unique signature. Paired with MHC II, which can be highly expressed by DCs and B cells.
	BUV496	CD8	Low brightness - low impact on other fluorochromes. Paired with CD8, which is highly expressed by T cells and can be co-expressed with drop-ins.
	BUV563	CD11c	Medium brightness - can impact PE detection. Paired with CD11c, which is only highly expressed by cDCs.
	BUV661	CD127	Medium to high brightness - can impact fluorochromes with the same wavelength emission due to cross-excitation. Paired with CD127, which is lowly expressed by subtypes of T cells.
	BUV737	F4/80	Medium to high brightness - can impact fluorochromes with the same wavelength emission due to cross-excitation. Paired with F4/80 because it is expressed only by macrophages and eosinophils.
	BUV805	CD3	Low brightness - low impact on other fluorochromes. Paired with CD3, which is expressed by all T cells and can be co-expressed with drop-ins.
Violet	BV421	**Drop-in**	High brightness and low similarity with other fluorochromes - ideal for lowly expressed markers.
	BV570	CD11b	Low brightness - low impact on other fluorochromes. Paired with CD11b, which is expressed by myeloid cells and can be co-expressed with other drop-ins.
	BV605	**Drop-in**	High brightness - it can impact other fluorochromes with the same wavelength emission. Appropriate for lowly expressed markers.
	BV650	B220	High brightness - can impact fluorochromes with the same wavelength emission due to cross-excitation. Paired with B220, which is uniquely expressed by B cells and pDCs
	BV711	Ly6G	High brightness - can impact fluorochromes with the same wavelength emission due to cross-excitation. Paired with Ly6G, which is only expressed by neutrophils.
	BV785	**Drop-in**	High brightness - it can impact other fluorochromes with the same wavelength emission. Good for lowly expressed markers.
Blue	BB515	**Drop-in**	High brightness and low similarity with other fluorochromes - ideal for lowly expressed markers.
	BB700	NK1.1	High brightness - can impact fluorochromes with the same wavelength emission due cross-excitation. Paired with NK1.1, which is expressed by NK cells.
	RB780	Ly6C	High brightness - unique signature. Paired with Ly6C, which can be dimly or highly expressed by monocytes and pDCs.
YG	PE	**Drop-in**	High brightness and large commercial availability.
	PE-Cy5	CD25	High brightness - can impact fluorochromes with the same wavelength emission due to cross-excitation. Paired with CD25 to identify Tregs.
	PE-Cy7	**Drop-in**	High brightness - it can impact other fluorochromes with the same wavelength emission. Good for lowly expressed markers.
Red	APC	**Drop-in**	High brightness and large commercial availability.
	R718	CD4	Medium to high brightness - low impact on APC and other fluorochromes. Paired with CD4, which can be co-expressed with drop-ins.
	APC-Fire780	CD45	Low to medium brightness - unique signature. Paired with CD45, which is co-expressed with all the other markers.
	Live Dead NIR	Dead cells	Has impact in many fluorophores, but is negative on live cells.

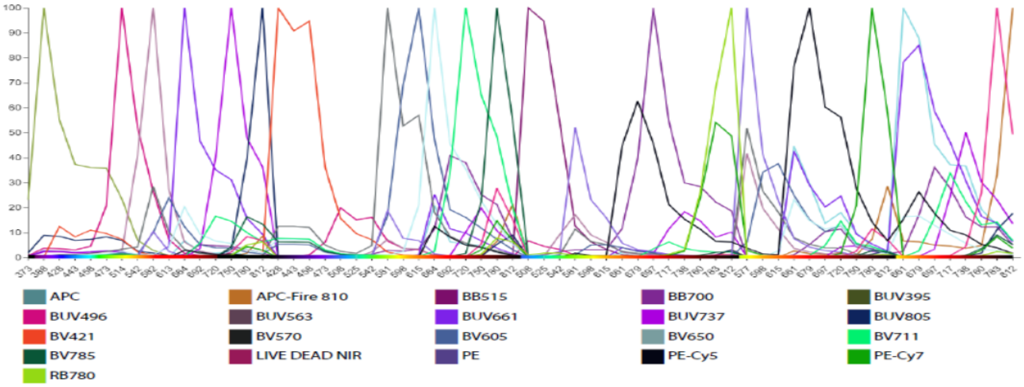

Supplementary Figure S2. The rationale for fluorochrome and marker pairing (above) and spectrum emission profile for all the backbone fluorochromes and the suggested drop-in fluorochromes. Spectrum emission curves were made using the Cytek® Full Spectrum Viewer tool (https://spectrum.cytekbio.com) (below).

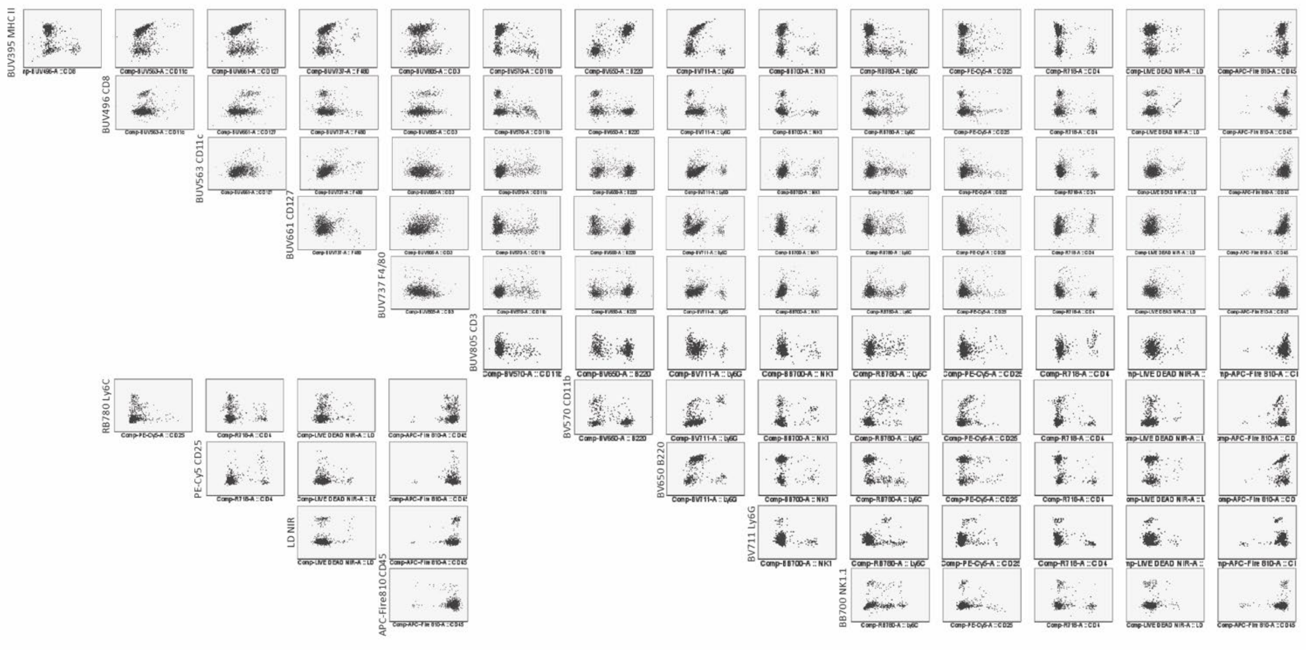

Supplementary Figure S3. Unmixing evaluation. Example of N x N plots showing spleen cells from C57B6/J mice stained with the backbone panel after applying the unmixing matrix. Cells were acquired on Cytek Aurora. N x N plots were generated using BD FlowJo software.

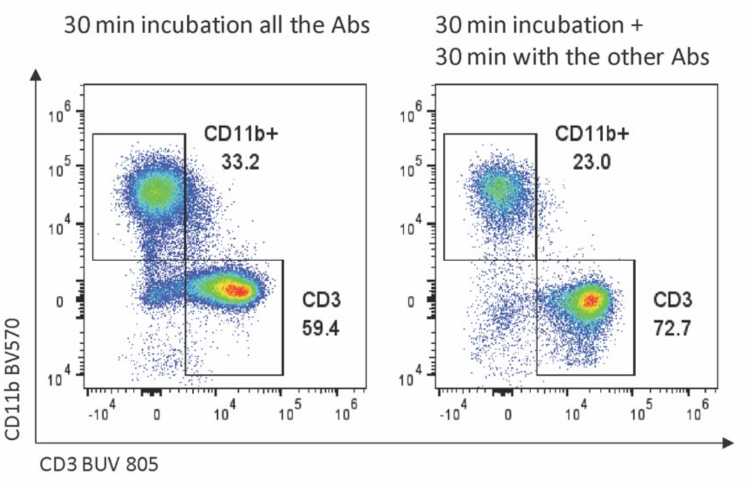

Supplementary Figure S4. Effect of prolonged incubation with the CD3 antibody (BUV805, clone 17A2). Pseudocolor plots showing the resolution of the $CD3^+$ positive population, staining with either the complete backbone cocktail for 30 min at 4°C, or first with the anti-CD3 antibody only for 30 minutes, followed by the addition of the remaining antibodies and a 30-minute incubation at 4°C. Splenocytes from C57BL/6J mice were used for this experiment.

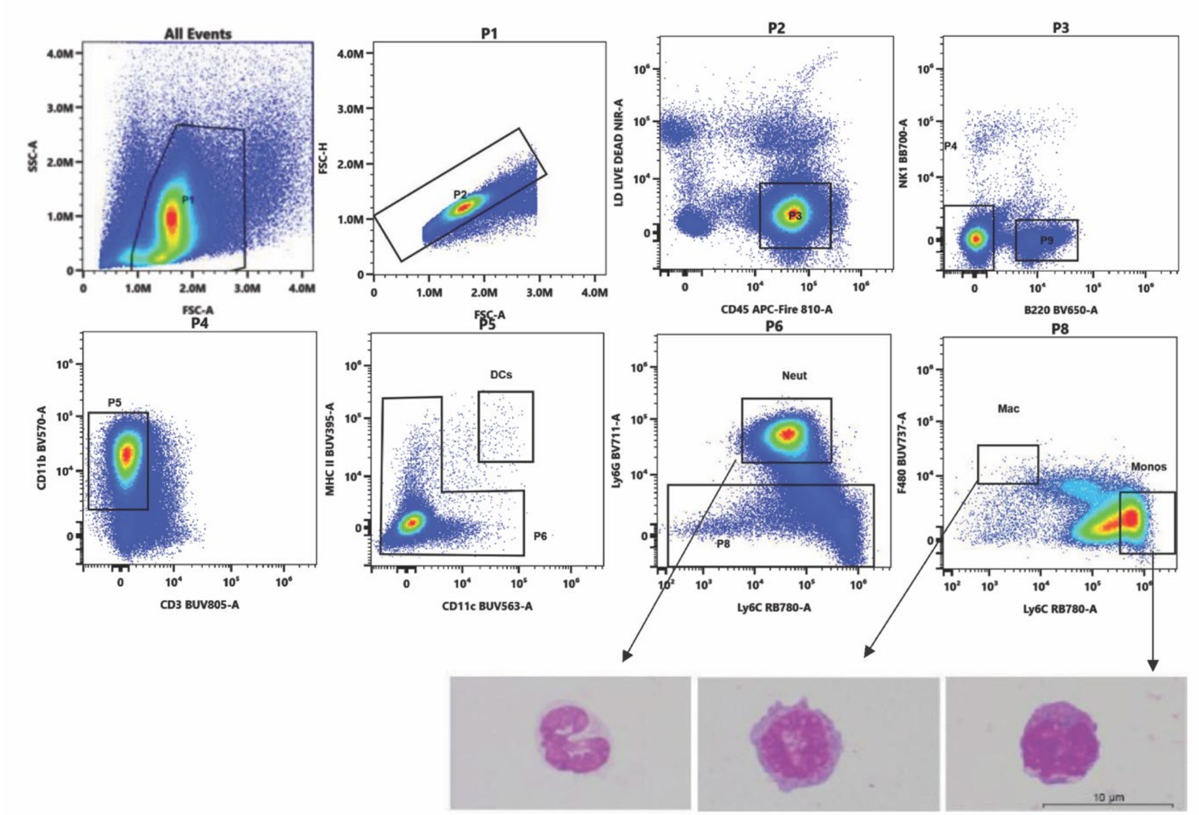

Supplementary Figure S5. Gating strategy for sorting immune cells from a spleen sample on Cytek Aurora CS. Splenocytes from C57BL/6J mice were stained with the backbone and sorted using Aurora CS. Representative images of a monocyte, a macrophage, and a neutrophil on Cytospin slides stained after using the Giemsa-Wright method are shown. Pictures were taken with oil lens (x100) and x10 eye piece, with a total magnification of x1000. Scale bar: 10 µm.

Drop-ins

Fluor.	Antigen
BV421	CD62L
BV605	Siglec-F
BV785	c-Kit
BB515	-------
FITC	CD44
PE	TIM-4
PE-Cy7	PD-1
APC	Lag-3

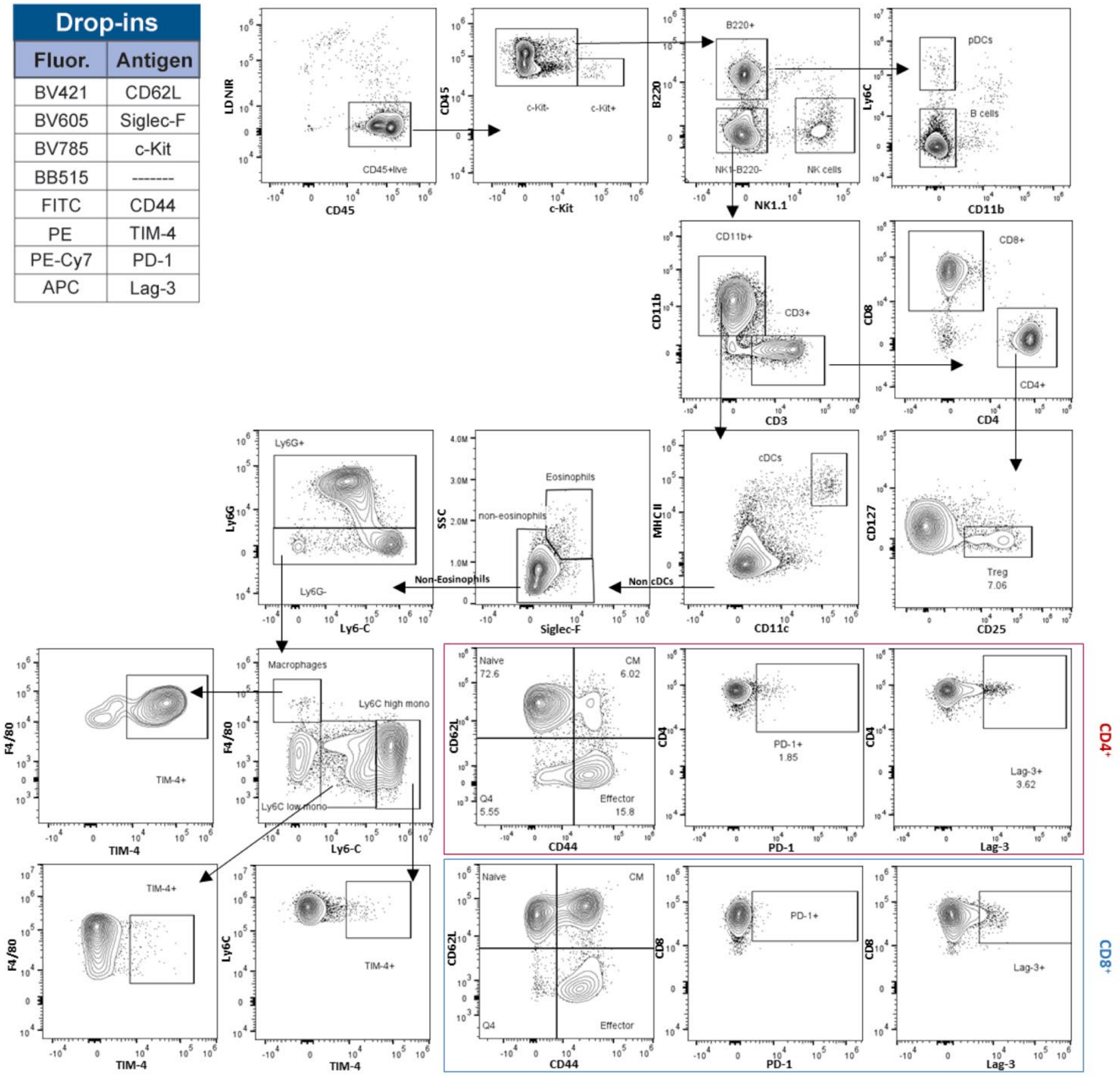

Supplementary Figure S6. Manual analysis of spleen cells stained with the immune cell panel consisting of both backbone and dedicated drop-ins. The main immune populations were defined, and immune checkpoint markers such as PD-1 and LAG-3 on T cells and TIM-4 on macrophages and monocytes were analyzed. Memory-effector cells were also analyzed for T cells. The cells were acquired on Cytek Aurora, and data were analyzed using BD FlowJo software.

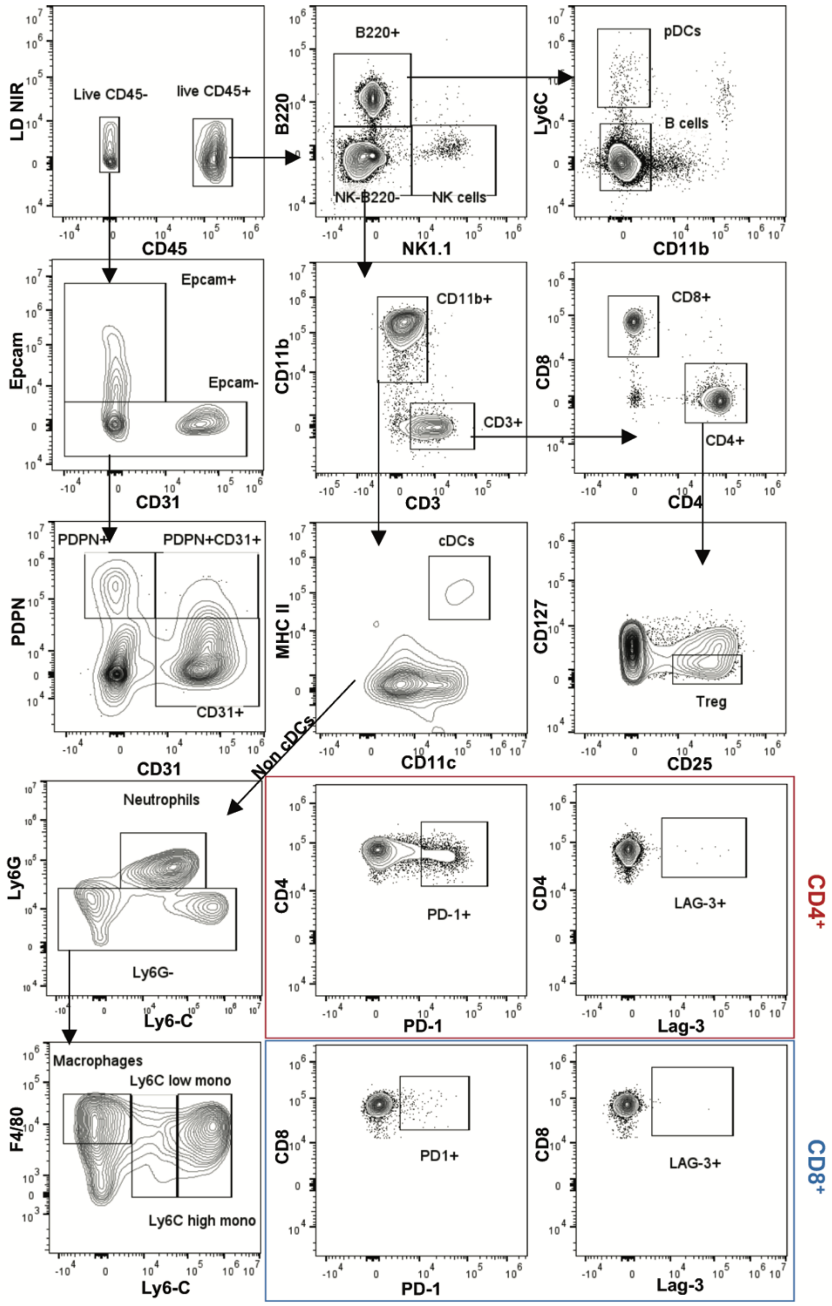

Supplementary Figure S7. Manual analysis of cells harvested from murine KRAS-driven lung adenocarcinoma. Manual analysis of pooled lung tumor single-cell suspension from mice with KRAS-driven lung adenocarcinoma generated by orthotopic transplantation (i.e., C57B6/N mice injected with *Kras*$^{G12C/+;}$ *Trp53*$^{fl/fl}$ lung cells from a syngeneic transgenic mouse) stained with the backbone panel plus drop-ins of the TME panel (i.e., Ep-CAM, CD31, PDPN, PD-1, and Lag-3). Cells were acquired on Cytek Aurora, and data were analyzed on BD FlowJo software.

Supplementary Table 1. Antibodies used to stain all the samples included in this study.

Fluor.	Marker	Clone	Manu.	Cat. #	Lot #	Ref. Ctrl.	Purpose	Panel	Conc. (mg/mL)
**BUV395**	MHCII	2G9	BD Biosciences	569244	2339103	Cells	cDCs	Backbone	1.25E-03
**BUV496**	CD8	53-6.7	Invitrogen	364-0081-82	2540613	Cells	$CD8^+$ T cells	Backbone	1.00E-02
**BUV563**	CD11c	HL3	BD Biosciences	749091	3220170	Cells	DCs	Backbone	2.00E-02
**BUV661**	CD127	A7R34	Invitrogen	376-1271-82	2681128	Beads	Tregs	Backbone	2.50E-03
**BUV737**	F4/80	T45-2342	BD Biosciences	749283	3213765	Cells	Macrophages	Backbone	1.25E-03
**BUV805**	CD3	17A2	Invitrogen	368-0032-80	2611752	Cells	T cells	Backbone	2.50-03
**BV421**	CD62L	MEL-14	BioLegend	104436	B391626	Beads	T cell activation	Immune/ TME	5.00E-03
**BV421**	Podoplanin	8.1.1	BioLegend	127423	B379391	Beads	Fibroblasts/ lymphatic vessels	TME	1.25E-03
**BV480**	FceR1	MAR-1	BD Biosciences	751769	2287323	Cells	Mast cells/ Basophils	Immune	2.50E-03
**BV570**	CD11b	M1/70	BioLegend	101233	B369858	Cells	Myeloid cells	Backbone	5.00E-03
**BV605**	Siglec-F	E50-2440	BD Biosciences	740388	3100371	Beads	Eosinophils	Immune	2.00E-03
**BV605**	Ep-CAM	G8.8	BioLegend	118227	B388536	Beads	Epithelial cells	TME	2.50E-03
**BV650**	B220	RA3-6B2	BioLegend	103241	B382458	Cells	B cells/pDCs	Backbone	1.25E-03
**BV711**	Ly6G	1A8	BD Biosciences	563979	2293297	Cells	Neutrophils	Backbone	5.00E-03
**BV785**	c-Kit	2B8	BD Biosciences	564012	2315764	Beads	Cancer cells/ stem and progenitor cells	Immune/ TME	1.25E-03
**BB515**	LAG-3	C9B7W	BD Biosciences	564672	2038083	Beads	T cell exhaustion	Immune/ TME	1.25E-03
**BB700**	NK1.1	PK136	BD Biosciences	566503	3095677	Beads	Natural killer cells	Backbone	5.00E-03
**RB780**	Ly6C	HK1.4 rMAb	BD Biosciences	755871	3051563	Cells	pDCs/ Monocytes	Backbone	2.50E-03
**PE**	Tim-4	RMT4-54	BioLegend	130006	B391498	Cells	T cell activation	Immune	1.00E-02
**PE-Cy5**	CD25	PC61.5	Invitrogen	15-0251-82	2616133	Beads	Tregs	Immune	6.25E-04
**PE-Cy7**	PD-1	RMP1-30	BioLegend	109109	B375843	Beads	T cell exhaustion	Immune/ TME	1.25E-03
**APC**	LAG-3	C9B7W	BioLegend	125210	B370710	Beads	T cell exhaustion	Immune/ TME	2.50E-03
**APC**	CD31	MEC13.3	BioLegend	102509	B398122	Beads	Endothelial cells	TME	6.20E-04
**RB718**	CD4	GK1.5	BD Biosciences	567311	2206862	Cells	$CD4^+$ T cells	Backbone	1.25E-03
**APC/ Fire 810**	CD45	30-F11	BioLegend	103173	B335318	Cells	Hematopoietic cells	Backbone	1.25E-03
**FITC**	CD44	IM7	BioLegend	103022	B378954	Beads	T cell activation	Backbone	2.50E-03
